# Identification and validation of two quantitative trait loci for dwarf bunt in the resistant cultivar ‘UI Silver’

**DOI:** 10.1007/s00122-024-04795-7

**Published:** 2025-01-07

**Authors:** Pabitra Joshi, Guriqbal Singh Dhillon, Yaotian Gao, Amandeep Kaur, Justin Wheeler, Xianming Chen, William Krause, Margaret R. Krause, Jianli Chen

**Affiliations:** 1 Department of Plant Sciences, University of Idaho Aberdeen, R and E Center, Aberdeen, ID 83210 USA; 2 Department of Plant Pathology, USDA-ARS, Wheat Health, Genetics, and Quality Research Unit and, Washington State University, Pullman, WA 99164–6430 USA; 3 Department of Crop and Soil Science, Oregon State University, Corvallis, OR 97331 USA

## Abstract

**Key message:**

Two dwarf bunt resistance QTLs were mapped to chromosome 6D, and KASP markers associated with the loci were developed and validated in a panel of regionally adapted winter wheats. UI Silver is an invaluable adapted resistant cultivar possessing the two identified QTL potentially associated with genes *Bt9* and *Bt10* and will be useful in future cultivar development to improve dwarf bunt resistance.

**Abstract:**

Dwarf bunt, caused by *Tilletia controversa*, is a fungal disease of wheat that can cause complete loss of grain yield and quality during epidemics. Traditional breeding for dwarf bunt resistance requires many years of field screening under stringent conditions with disease assessment possible only near or after plant maturity. Molecular marker-assisted selection (MAS) offers a more efficient alternative. This study identified quantitative trait loci (QTL) and associated molecular markers for dwarf bunt resistance in wheat. A doubled haploid (DH) mapping population of 135 lines, derived from bunt-resistant cultivar ‘UI Silver’ and susceptible line ‘Shaan89150’, was evaluated in field nursery in Logan, Utah in 2017, 2018, and 2023. The population was genotyped using Illumina 90 K SNP iSelect marker platform. Using inclusive composite interval mapping (ICIM), the major QTL *Qdb.ssdhui-6DL* was consistently identified on chromosome arm 6DL across all environments, explaining phenotypic variations ranging from 15.29% to 35.40%. Another QTL, *Qdb.ssdhui-6DS*, was detected on chromosome arm 6DS, explaining approximately 11% of the phenotypic variation. These two QTLs exhibit additive-by-additive effects for increased resistance within the DH population. Kompetitive allele-specific PCR (KASP) markers were developed within QTL intervals and used in a validation panel of regionally adapted winter wheat lines to confirm the association between the two QTL and dwarf bunt resistance. Thus, ‘UI Silver’ and additional resistant cultivars with these two QTLs are valuable parental lines for improving dwarf bunt resistance through marker-assisted selection. These genetic resources are essential for understanding gene function via map-based gene cloning.

**Supplementary Information:**

The online version contains supplementary material available at 10.1007/s00122-024-04795-7.

## Introduction

The global human population is growing rapidly and is expected to reach 9 billion by 2050 (Ray et al. 2013). This increase in population will result in a higher demand for food. Bread wheat (*Triticum aestivum* L., 2n = 6x = 42, AABBDD) is one of the world’s major food crops, providing 20% of the calories and 25% of the protein consumed in the human diet (Alqudah et al. 2020). However, wheat production faces many challenges including climate change, limited natural resources such as water and arable land, and escalating biotic and abiotic stresses. These challenges have raised concerns about the sustainability of wheat production and have highlighted the urgent need for innovative solutions to enhance yield and resilience (Hunter et al. 2017).

Among the biotic stresses, dwarf bunt (DB), caused by *Tilletia controversa* Kühn, poses a significant threat to wheat, particularly in dryland production areas with prolonged snow cover (Goates 1996). Recent estimates indicate that DB caused yield losses totaling 1,018,054 bushels (16,967.57 tons) in the USA from 2018 to 2022, with Idaho being one of the most affected states (Crop Protection Network 2024). Additionally, quarantine restrictions due to DB presence significantly hinder international wheat trade, as demonstrated by China’s strict import regulations from DB-affected areas (Ren et al. 2021).

Common bunt (CB), caused by the fungal pathogens *T. caries* and *T. laevis*, is also a threat to wheat production and its biology is similar to that of DB (Goates 2012; Muellner et al. 2021; Ehn et al. 2022). CB epidemics frequently occur in winter wheat production areas in Europe and spring wheat production areas in Canada, leading to yield reductions and decreased market value of contaminated seed (Goates 1996; Aydoğdu and Kaya 2020; Lunzer et al. 2023b). In the USA, CB infections resulted in losses totaling 717,873 bushels (11,964.55 tons) from 2018 to 2022 (Crop Protection Network 2024).

Teliospores are the primary inoculum for both bunt diseases, with teliospores of the DB pathogen having prolonged viability in soil, thereby making disease management particularly challenging (Tyler and Jensen 1958; Goates 1996; Borgen and Davanlou 2001). Managing DB is especially difficult in organic wheat production, where the use of chemical fungicides is prohibited. As a result, alternative control methods must be used such as biological control agents, which are often more costly and less effective (Matanguihan et al. 2011; Muellner et al. 2021). Conventional farming systems also face escalating costs and potential shortages of chemical fungicides, highlighting the need for sustainable disease management practices across all agricultural contexts (Steffan et al. 2017). Developing genetic resistance to bunt diseases is a sustainable and effective solution to meet these challenges (Wang et al. 2019; Gordon et al. 2020).

Traditionally, resistance to bunt diseases has been understood as a qualitative trait controlled by major resistance genes (R genes) following the gene-for-gene concept (Flor 1933, 1971). Researchers have postulated seventeen race-specific resistance genes (*Bt1*-*Bt15*, *Btp*, and *Bt-unknown*) based on phenotypic evaluation of differential lines (Goates 1996, 2012). These genes enable monitoring virulence shifts within fungal populations (Liatukas and Ruzgas 2008; Wang et al. 2019; Gordon et al. 2020; Muellner et al. 2021; Lunzer et al. 2023a). Using the differential lines derived bi-parental populations, *Bt9* (Steffan et al. 2017), *Bt10* (Menzies et al. 2006), and *Bt12* (Muellner et al. 2020) have been genetically mapped.Single QTL on 6DL, 6DS, and 7DS were associated with genes *Bt9*, *Bt10*, and *Bt12*, respectively. While these QTL studies provided an important basis for understand the genetics of resistance, they were limited in both scope and application as all studies were based on resistance to CB, and no diagnostic molecular markers were either developed or validated in regionally adapted breeding lines or released resistant winter wheat cultivars.

Various mapping populations and association panels have been used to identify QTLs for CB resistance in wheat. These QTLs span many chromosomes, including 1A, 1B, 2A, 2B, 3AL, 3BS, 3DL, 4AL, 4BS, 4DS, 5AL, 5BS, 5BL, 5DL, 6AL, 6BL, 6DS, 7AL, 7AS, 7BS, and 7DL (Menzies et al. 2006; Fofana et al. 2008; Wang et al. 2009; Dumalasová et al. 2012; Knox et al. 2013; Singh et al. 2016; Steffan et al. 2017; Zou et al. 2017; Bhatta et al. 2018; Mourad et al. 2018; Muellner et al. 2021; Ehn et al. 2022; Lunzer et al. 2023a). This widespread distribution across the wheat genome suggests a complex genetic architecture for CB resistance. Far fewer studies of DB resistance have been performed, primarily due to the stringent environmental requirements for disease development. QTLs for DB have been located on chromosomes 1AL, 2BS, 6DL, 7AL, and 7DS. (Chen et al. 2016; Wang et al. 2019; Gordon et al. 2020; Muellner et al. 2021). Among these, a QTL on chromosome 6DL has been consistently identified in several study across multiple mapping populations and environments, suggesting its potential for marker-assisted selection (MAS) in wheat breeding programs (Steffan et al. 2017; Wang et al. 2019; Lunzer et al. 2023a).

Previous QTL mapping studies for bunt resistance have often used landraces and unadapted resistant lines (Chen et al. 2016; Wang et al. 2019; Gordon et al. 2020; Muellner et al. 2021). The limited validation in adapted breeding lines and released resistant cultivars has hindered the application of MAS for bunt resistance. The present study validated previously identified QTLs in a bi-parental population and in a panel of regionally adapted winter wheat. The bi-parental population was derived from a cross between a widely grown resistant cultivar ‘UI Silver’ and the susceptible line ‘Shaan89150’. The validation panel consisted of adapted lines primarily from breeding programs at Utah State University (USU), the University of Idaho (UI), and Washington State University (WSU). DB resistance has been a primary goal of the USU and UI winter wheat breeding programs. Both populations were phenotyped for DB resistance in the field and genotyped using the Illumina 90 K SNP iSelect marker platform. The bi-parental population was used for QTL identification, while the validation panel was used for QTL and marker validation.

## Materials and methods

### Plant materials

‘UI Silver’ (PI 658467, PVP 201400011) is a hard white winter wheat cultivar released in 2009 and adapted primarily in dryland production conditions with resistance to DB, adult plant resistance to stripe rust (*Puccinia striiformis* f. sp. *tritici*), and resistance to some races of stem rust (*Puccinia graminis*) (https://npgsweb.ars-grin.gov/gringlobal/accessiondetail?id=1825466). UI Silver has shown excellent DB resistance since its release in 2009 and has been used as a check cultivar for bread-baking quality for the hard white winter wheat market (Chen, personal communication). To better understand the genetic control of DB resistance in UI Silver, a mapping population (henceforth referred to as SSDH), comprised of 135 lines, was developed from a cross between UI Silver and a susceptible line ‘Shaan89150’ using the wheat × maize doubled haploid method (Laurie and Bennett 1986). Shaan89150 is a germplasm line originally developed by the Northwest Agricultural University in China with good stripe rust resistance. It was found susceptible to DB when evaluated in the USU dwarf bunt nursery in Logan, Utah (Chen, personal communication).

The validation panel consisted of 175 winter wheat lines, primarily from three breeding programs in the Pacific Northwest (PNW): USU, the UI, and WSU. The pedigree, market class, and origin of the lines in the panel are included in Table S1. The panel was used to assess the QTLs identified in the SSDH population.

### Assessment of DB resistance in field experiments

The DB screening nursery located at the USU Research Farm in Logan, Utah (41°45′46.46″N, 111°48′54.98″W, elevation: 1400 m), consistently produces high DB disease pressure due to the frequent occurrence of extended snow cover conditions, which is a critical factor for *T. controversa* spore germination (Muellner et al. 2021). Breeding lines from the Intermountain West and abroad have been screened at this nursery for several decades (Hole and Clawson 2023).

The SSDH population was assessed in Logan nursery during three growing seasons in 2017, 2018, and 2023 (designated as DB17, DB18, and DB23, respectively). The validation panel was assessed in the Logan nursery in 2022 and 2023 (designated as DB22 and DB23 respectively). All lines in the two populations were sown in 1 m headrows, and the trials were laid out as randomized complete block design with two replications (blocks) (Wang et al. 2019; Muellner et al. 2021).

The DB nursery was inoculated with a water suspension of *T. controversa* teliospores after seedling emergence but before snow cover (Table S2). The inoculum used originated from diseased spikes collected within the DB nursery during the previous year. Spikes with visible bunt sori were ground and mixed in water, and the suspension was filtered through cheesecloth. The suspension was diluted to a target concentration of 2–3 million teliospores per ml of water. Individual rows were inoculated in late November using approximately 100 ml of the spore suspension per meter, aiming for a final application of 200–300 million spores per 1 m row (Wang et al. 2019). At plant maturity (Zadoks stage 92; Zadoks et al. 1974), the total number of spikes and the number of infected spikes were recorded for each row. A spike was considered infected if it contained at least one bunted spikelet (Goates 2012). DB disease incidence (DBI) was then calculated for each row as the percentage of spikes with bunt-infected spikelets.

### Phenotypic analysis

Within each year (*i.e.*, environment), the mean DBI was calculated for each line in the SSDH and validation populations. An analysis of variance (ANOVA) was performed to compare DBI across the three years of evaluation (DB17, DB18, DB23). The model included year, genotype (DH lines), and the year-by-genotype interaction as factors. Tukey’s HSD test was applied to assess pairwise differences between years. All statistical analyses were conducted using R software environment (R version 4.3.2, http://www.r-project.org). Best linear unbiased predictors (BLUPs) of DBI were calculated for each line in the SSDH and validation populations as adjusted means across environments. The terms for genotypes, environments (*i.e.*, nursery years), and replicates were fit as random using Meta-R (Alvarado et al. 2020).

1 $$Y_{ijk} = \mu + G_{i} + E_{j} + R_{k\left( j \right)} + + EG_{ij} + \varepsilon_{ijk}$$

where Y_ijk_ is the DBI trait value, μ is the mean effect, G_i_ is the effect of the *i*^*th*^ genotype, E_j_ is the effect of *j*^*th*^ environment, R_k(j)_ is the effect of the *k*^*th*^ replicate in the *j*^*th*^ environment, EG_ij_ is the effect of the interaction between the *i*^*th*^ genotype and the *j*^*th*^ environment, and ε_ijk_ is the error associated with the *i*^*th*^ genotype, the *j*^*th*^ environment, and the *k*^*th*^ replicate. All random effects were assumed to be normally and independently distributed with mean zero and variance σ^2^. Broad sense heritability (H^2^) was estimated with the equation:

2 $$H^{2} = \frac{{\sigma_{G}^{2} }}{{\sigma_{G}^{2} + \frac{{\sigma_{GE}^{2} }}{e} + \frac{{\sigma_{\varepsilon }^{2} }}{er}}}$$

where σ^2^_G_ is the variance of genotypes, σ^2^_GE_ is the variance of the genotype–environment (i.e., trial) interaction, σ^2^_ε_ is the residual variance, *e* represents the number of environments (i.e., trials), and *r* represents the number of replicates in each environment (i.e., trial). Correlation coefficients among different trials were calculated using the “corrplot” package (Wei and Simko 2021) for the R software environment (R version 4.3.2, http://www.r-project.org).

### Genotypic analysis

Genomic DNA was extracted from the DH and parental lines using the CTAB method (Saghai-Maroof et al. 1984). The lines were then genotyped with the Wheat 90 K SNP Illumina iSelect platform (Wang et al. 2014) by the USDA-ARS Small Grains Genotyping Laboratory in Fargo, ND. SNP calling was performed using Genome Studio 2.0 with the Polyploid Clustering Module v1.0 developed by the platform manufacturer Illumina (San Diego, CA) (Illumina 2010). For quality control, the raw SNP data were filtered such that any markers that did not show polymorphism between the two parents were excluded. Further filtration was performed to exclude markers with at least 20% missing data and 10% segregation distortion. The raw 90 K SNP data generated during genotyping are provided as Supplementary File 2.

### Linkage map construction

For linkage map construction, co-segregated markers were first identified and excluded, retaining only one randomly selected SNP for the purpose of mapping. Linkage analysis was conducted in a two-step process using the JoinMap® 4.0 software (Ooijen et al. 2006). A map was constructed using the Kosambi mapping function (Kosambi 1943) with a minimum logarithm of odds (LOD) score of 7.0. A regression mapping algorithm was then used to determine the order of markers within each linkage group and calculate the genetic distances between them. The linkage groups were numbered by default with the descending numbers of markers present in each linkage group. Linkage groups were further divided if the genetic distance between two adjacent markers exceeded 50 centimorgans (cM) (Wang et al. 2019). Finally, the R package “ggplot2” (Wickham 2016) was used to visualize potential synteny with the Chinese Spring reference genome (International Wheat Genome Sequencing Consortium RefSeq v2.1) (Zhu et al. 2021). Synteny refers to the conservation of gene order between linkage groups and their corresponding physical locations on chromosomes.

### QTL analysis

DBI phenotypes of the SSDH lines evaluated in DB17, DB18, and DB23, and BLUPs calculated across years were used for QTL analysis. QTLs were detected using inclusive composite interval mapping (ICIM) with QTL IciMapping 4.1 software (https://isbreeding.caas.cn/rj/index.htm). This software employs a stepwise regression approach that considers all marker information simultaneously (http://www.isbreeding.net/). The parameters used for QTL mapping included a walking speed of 0.01 centimorgans (cM) and a p-value threshold for inclusion of 0.001. The significance of identified QTLs was evaluated using 1000 permutations to establish a threshold LOD score with a type I error rate of 0.05. The QTL IciMapping software provided the proportion of the phenotypic variance explained (PVE%) by each QTL and the magnitude of their additive effects.

The effect of identified QTLs on DB disease resistance was further investigated using eta-squared designated as η^2^ to quantify the proportion of the phenotypic variance explained by each QTL. This index ranges from 0 to 1, with higher values indicating a greater proportion of the variation in DBI phenotype explained by the QTL (Tomczak and Tomczak-Łukaszewska 2014). The following equation was used to calculate eta-squared (3):

3 $$\eta^{2} = \left( {H - k + 1} \right)/\left( {n - k} \right)$$

where H represents the Kruskal–Wallis test statistic, k is the number of alternative alleles at the QTL, and n is the total number of observations. Effect sizes based on η^2^ value were categorized as small (0.01 to < 0.06), moderate (0.06 to < 0.14), and large (≥ 0.14) based on the H value obtained from Kruskal-Wallis test statistic (Tomczak and Tomczak-Łukaszewska 2014). Finally, the differences in DBI among genotypes grouped by QTL combination were compared by conducting one-way ANOVA with Tukey’s HSD at *p* < 0.001. These were plotted as boxplots using the “ggplot2” package for R (Wickham 2016). To test for interaction effects between the identified QTLs, a two-way ANOVA was conducted using the peak marker data. The ANOVA tested for both the main effects of each QTL and the interaction between them, with the response variable being the BLUPs calculated across years. The significance of the interaction term was reported.

## KASP marker design and genotyping.

The polymorphic SNPs that were located within the confidence intervals of the identified QTLs were converted to Kompetitive allele-specific PCR (KASP) primers using the Polymarker software (Ramirez-Gonzalez et al. 2015). Most of the KASP markers were designed based on sequences available from the Polymarker website (https://www.polymarker.info/), which was originally developed for the 90K SNP Illumina iSelect array. However, some markers that did not perform optimally were re-designed for improved efficacy. To characterize the KASP markers in the validation population, DNA was extracted from each line using the CTAB method (Saghai-Maroof et al. 1984). The KASP assays were performed on a CFX384 Touch™ Real-Time PCR Detection System (Bio-Rad, Hercules, CA) with a reaction volume of 5.07 µl. The reaction mix contained 2.5 µl of KASP Master Mix, 0.07 µl of the KASP primer mix, and 2.5 µl of genomic DNA at a concentration of 50 nanograms per microliter. The KASP assays were conducted according to the manufacturer’s instructions (http://www.lgcgroup.com). Following amplification, the genotyping data were visualized and analyzed using the allelic discrimination function available within the CFX Maestro software (Bio-Rad, Hercules, CA). This process was used to genotype the lines in the validation population for the targeted QTL regions and to verify their applicability of the associated KASP markers for use in MAS and gene pyramiding.

### QTL validation

The genotype data from the KASP assay of the validation panel were utilized to assess the impact of the identified QTLs on DB resistance (Table S6, S9). For validation, only the peak KASP marker data corresponding to each QTL were considered. The validation panel was then divided into four categories according to the presence or absence of the KASP marker allele associated with the peak SNP at each QTL (Table S6, S9). Eta-squared (η^2^) quantified the proportion of DBI phenotypic variance explained by each QTL (Tomczak and Tomczak-Łukaszewska 2014). Finally, a one-way ANOVA with Tukey’s HSD test (*p* < 0.05) was conducted to compare the differences in mean DBI among genotypes grouped by QTL combinations. The results were visualized as boxplots using the “ggplot2” package for R (Wickham 2016).

## Results

### Phenotypic variation within and across years

**Fig. 1:**
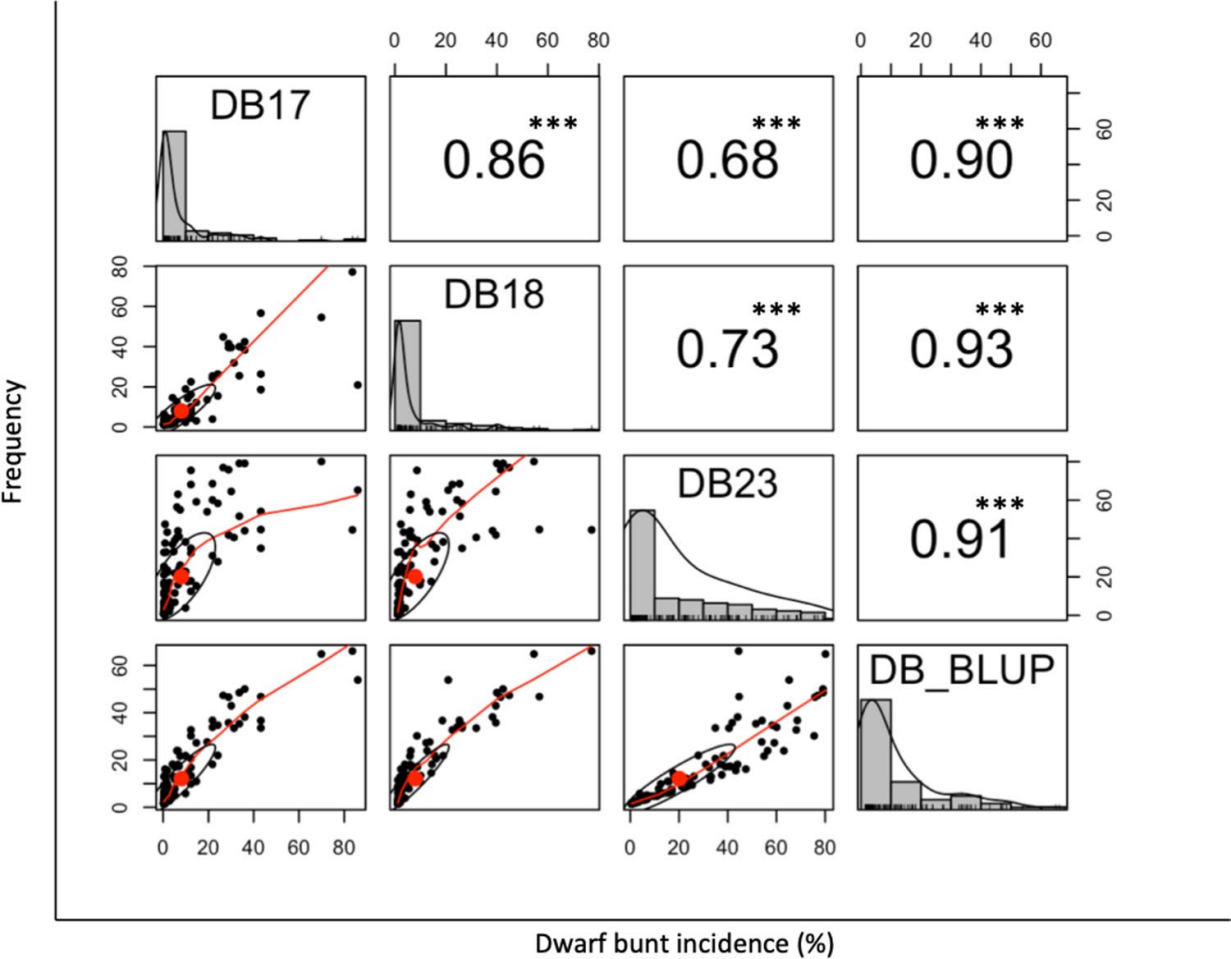
Distribution and correlation of dwarf bunt incidence (DBI%) in the SSDH population in each individual year and across-year DBI BLUPs. The diagonal contains histograms of DBI in each year. The lower diagonal contains scatterplots with a Lowess smoothing line between each year. The upper diagonal contains the Spearman’s rank correlation coefficient with significance test (*** indicates significance *p* < 0.001)

The DBI data showed a skewed distribution. A Shapiro–Wilk normality test (Shapiro and Wilk 1965) on the trial residuals indicated a significant deviation from normality (*p* < 0.0001). Similarly, logarithm base-10 transformations of the trials also showed significant deviations from normality (*p* < 0.0001). As a result, untransformed DB data were used for all subsequent analyses to maintain consistency in the statistical evaluations. This skewed distribution in all years suggests major gene effects within the population (Fig. 1 and Table 1). UI Silver was highly resistant with near zero DBI ranging from 0.40–1.59% across years, while the susceptible parent showed much higher DBI ranging from 30.11 to 64.51% (Table 1). The average DBI varied across years and was 7.99, 7.78, and 19.98% for DB17, DB18, and DB22 (Table 1). Seventy resistant lines showed DBI less than 10% in each of the three years with mean values of less than 5% (Table S1). The ANOVA revealed significant differences in DBI% across the three years (*p* < 0.0001) (Table S3). The mean DBI in 2023 was significantly higher than in 2017 and 2018 (Tukey’s HSD, *p* < 0.0001), while no significant difference was observed between 2017 and 2018 (Table S4). The genotypic effect was also significant (*p* < 0.0001), indicating variability in resistance among the DH lines (Table S3). High and significant Spearman’s rank correlation coefficients between individual year means and across-year BLUPs for DBI were observed, ranging from 0.68 to 0.93 at a significance level of *p* < 0.05, indicating trait stability across years (Fig. 1). The high H^2^ observed at 0.87 indicates that genotype was the primary source of the phenotypic variance (Table 1).

**Table 1:** Dwarf bunt incidence (DBI%) of each doubled haploid (DH) parent and the 135 DH lines tested over three years, the best linear unbiased predictors (BLUPs) across trials, and the broad sense heritability (H^2^) of DBI%

Field trial	Parents (DBI %)		DH Lines (DBI %)				H^2^
	Shaan89150	UI Silver	Min	Median	Max	Mean	
DB17	30.11	0.40	0.40	1.35	85.97	7.99	0.95
DB18	39.57	1.16	1.16	1.93	77.13	7.78	0.86
DB23	64.51	0.81	0.58	8.31	80.12	19.98	0.97
DB_BLUP	42.91	1.59	1.52	4.70	66.10	11.93	0.87

### Linkage mapping and QTL analysis

In the construction of the linkage map and subsequent QTL analysis, 1414 unique SNP markers were used, forming 34 linkage groups representing all wheat 21 chromosomes with a cumulative map length of 4474.9 cM (Table S5, S7). The largest linkage group corresponded to chromosome 2B, spanning a total length of 294.1 cM (Table S7). While most of the linkage groups represented complete wheat chromosomes, some chromosomes were divided into multiple linkage groups when distances between adjacent markers exceeded 50 cM (Table S5, S7).

**Fig. 2:**
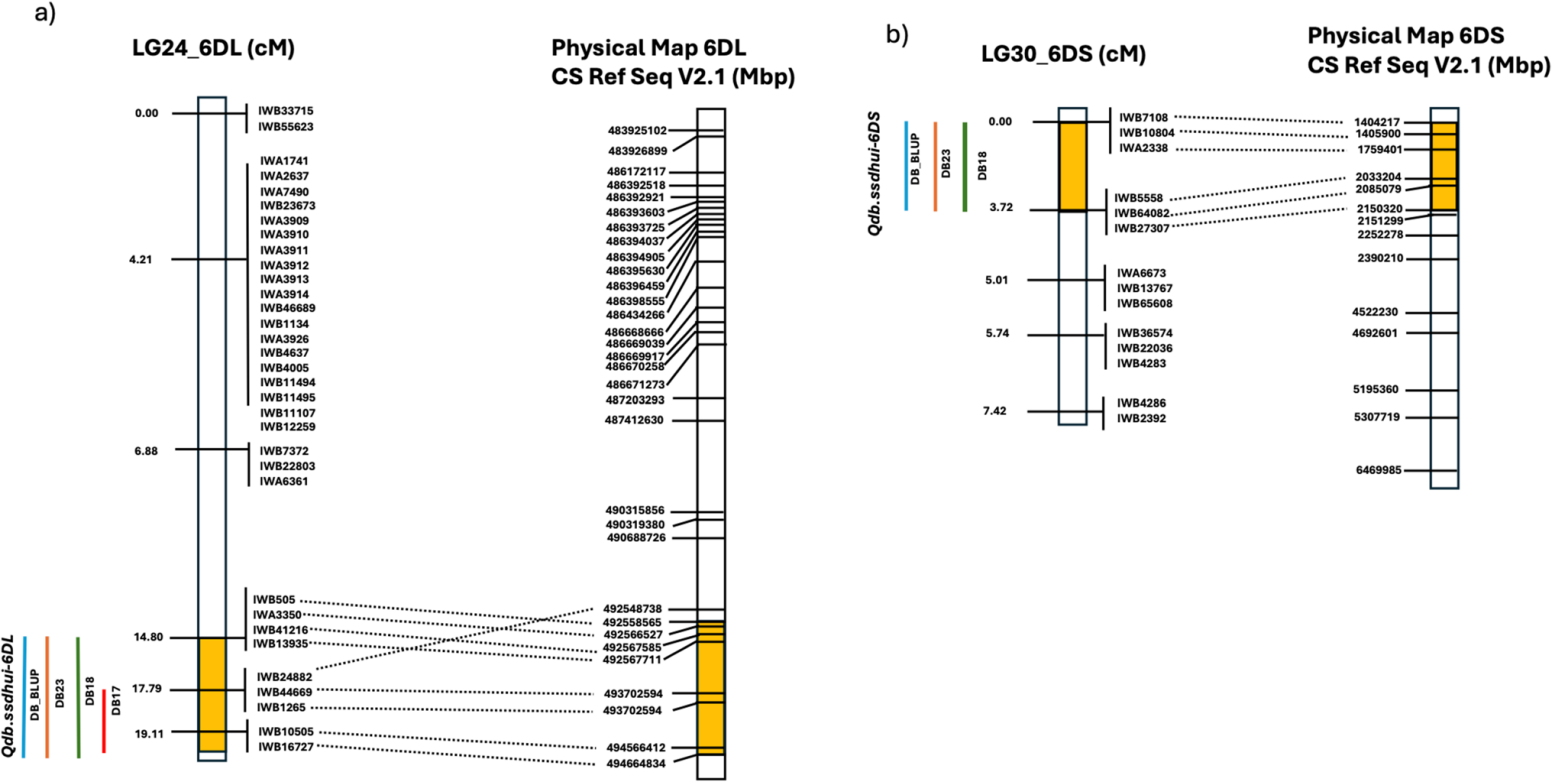
Genetic map (cM) and corresponding physical map (Mbp) based on the Chinese Spring (CS) reference sequence v2.1, showing the identified dwarf bunt resistance QTL **a** Qdb.ssdhui-6DL and **b** Qdb.ssdhui-6DS. The highlighted yellow regions indicate the QTL locations on both genetic and physical maps. Different colored lines represent individual years in which the QTL were detected. Co-segregating markers are included in the map

Two QTLs were identified with the ICIM method, with one on the long arm of chromosome 6D (*Qdb.ssdhui-6DL*) and another on the short arm of 6D (*Qdb.ssdhui-6DS*) (Fig. 2; Table 2). QTL *Qdb.ssdhui-6DL* was detected in all three years and using the across-year BLUPs, with a maximum LOD score of 14.4 (Table 2). QTL *Qdb.ssdhui-6DS* was detected in two of three years (undetected in DB17) and using the across-year BLUPs with a maximum LOD score of 5.3. *Qdb.ssdhui-6DL* explained 15.2 to 35.4% of the phenotypic variation, while QTL *Qdb.ssdhui-6DS* explained 10.89 to 12.14% of the phenotypic variation when considering the individual years and the across-year BLUPs (Table 2).

**Table 2:** Significant QTL identified in the SSDH population for dwarf bunt incidence (DBI%). QTL detection was performed for each of the three years (DB17, DB18, DB23) and using across-year best linear unbiased predictors (DB_BLUPs)

QTL	Year	QTL	QTL interval (cM)	Physical position (Mbp)	Peak position (cM)	Peak marker	LOD	PVE(%)	Effect
*Qdb.ssdhui-6DL*	DB17	6DL	17.7 − 19.1	492.5 − 494.6	17.7	IWB1265	5.4	17.0	6.1
	DB18	6DL	15.9 − 17.7	492.5 − 494.6	15.9	IWB41216	5.2	15.2	4.7
	DB23	6DL	16.4 − 17.7	492.5 − 494.6	16.4	IWB41216	14.4	35.4	13.5
	DB_BLUP	6DL	16.3 − 17.7	492.5 − 494.6	16.3	IWB41216	10.6	27.5	7.1
*Qdb.ssdhui-6DS*	DB18	6DS	0 − 3.7	1.4 − 2.1	1.0	IWB1708	3.9	11.1	4.0
	DB23	6DS	0 − 3.7	1.4 − 2.1	1.0	IWB1708	5.3	10.8	7.4
	DB_BLUP	6DS	0 − 3.7	1.4 − 2.1	1.0	IWB1708	5.2	12.1	4.7

### QTL effects and QTL by QTL interaction

**Fig. 3:**
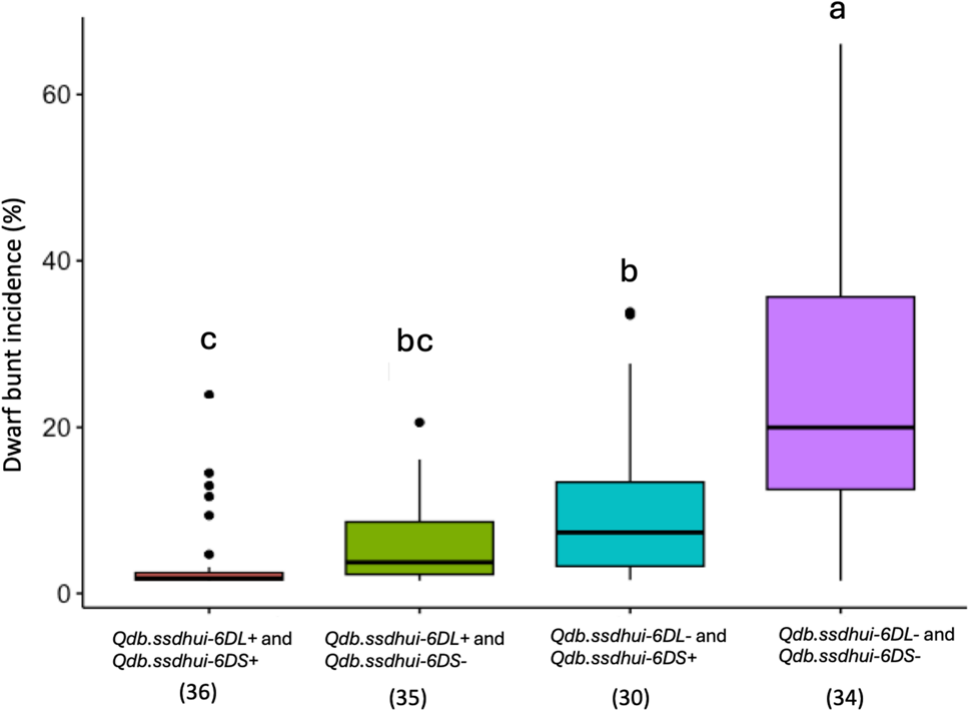
Effect of the two identified QTL on dwarf bunt incidence (DBI%) in the SSDH population using best linear unbiased predictors (BLUPs) across three years. The vertical axis represents DBI%. The box boundaries represent the interquartile range (IQR), with the lower and upper quartiles corresponding to the bottom and top of the box, respectively. The horizontal line within each box represents the median. Whiskers (lines extending from the box) indicate the range of data within 1.5 times the IQR, and data points outside this range are plotted as individual outliers (represented by dots). Means with different letters are significantly different (*p* < 0.0001), and sample sizes are shown in parentheses

DBI comparison showed that the lines with susceptible alleles at the identified QTL were highly susceptible with a mean DBI of 23.96% (Fig. 3). The presence of the QTL *Qdb.ssdhui-6DS* on chromosome 6DS alone imparted partial resistance to DB, with a mean DBI of 11.80%. The presence of the *Qdb.ssdhui-6DL* QTL alone on chromosome 6DL produced an even greater and significant reduction in DBI (at p < 0.001), with a mean of 8.75% (Fig. 3). Thus, the resistance effect of QTL *Qdb.ssdhui-6DL* is possibly greater than that of *Qdb.ssdhui-6DS* QTL. Lines with both the *Qdb.ssdhui-6DL* and *Qdb.ssdhui-6DS* QTLs had the lowest DBI of 3.74%, suggesting an additive effect of the two QTL (Fig. 3).

Effect size analysis showed that QTL *Qdb.ssdhui-6DL* had a larger effect (η^2^ = 25.8%) compared to QTL *Qdb.ssdhui-6DS* (η^2^ = 13.8%), which had a moderate effect (Table 3). The combined effect of both QTL (η^2^ = 46.4%) (Table 3) was greater than the effect of either QTL individually, supporting the hypothesis that the two QTL contribute additively to increased disease resistance.

**Table 3:** Effect of the two identified QTL on dwarf bunt incidence (DBI%) in the SSDH population using best linear unbiased predictors (BLUPs) across the three years with significant difference at a *p*-value of 0.05

QTL	H-statistic	Effect ( η^2^)	Magnitude
*Qdb.ssdhui-6DS*	9.57	0.138**	Moderate
*Qdb.ssdhui-6DL*	18.3	0.258***	Large
*Qdb.ssdhui-6DL: Qdb.ssdhui-6DS*	32.5	0.464***	Large

Level of significance; *p*-value < 0.001 (***), *p*-value < 0.01 (**), *p*-value < 0.05 ‘*’, *p*-value < 0.1 ‘.’

Further ANOVA using peak marker data showed that the interaction effects between *Qdb.ssdhui-6DL* and *Qdb.ssdhui-6DS* approached significance (*p* = 0.096) but did not meet the 0.05 threshold. The main effects of both QTL were significant (*p* < 0.001), suggesting that their effects are primarily additive (Table S8).

### Validation of the *Qdb.ssdhui-6DL* and *Qdb.ssdhui-6DS* QTLs

**Fig. 4:**
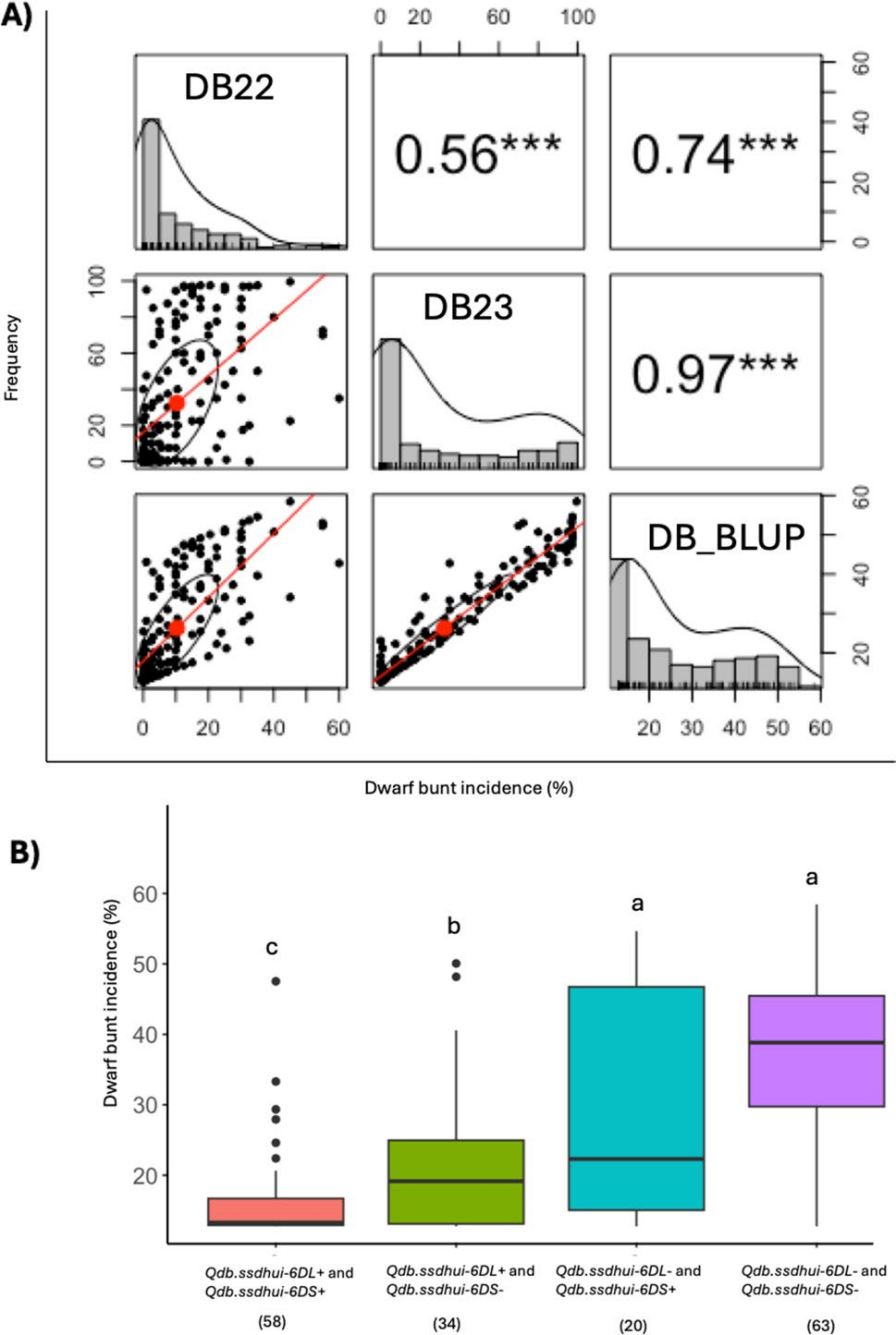
(A) Pairwise correlation analysis of dwarf bunt incidence (DBI %) in the validation population for two years (DB22, DB23) and for Best Linear Unbiased Predictors (BLUPs) across the two years. The upper panels show Pearson’s correlation coefficients, with significance levels indicated by asterisks (***p < 0.001). Histograms where the horizontal axis represents the DBI% and density plots for each variable are shown along the diagonal, while the lower panels display scatterplots with fitted regression lines. (B) Effect of the two identified QTLs on DBI% in the validation population using BLUPs across the two years. The vertical axis represents DBI%. The box boundaries represent the interquartile range (IQR), with the lower and upper quartiles corresponding to the bottom and top of the box, respectively. The horizontal line within each box represents the median. Whiskers indicate the range of data within 1.5 times the IQR, and data points outside this range are plotted as individual outliers (represented by dots). Means with different letters are significantly different (*p* < 0.0001), and sample sizes are shown in parentheses

The distribution of DBI in the validation population showed a left-skewed pattern with a bimodal tendency in DB23 and DB-BLUP (Fig. 4A). The average DBI was higher in 2023 (19.98%) than in 2022 (7.99%).

The genotype data from the KASP assay of the validation panel were analyzed to assess the influence of the identified QTL on DB resistance. Based on the presence or absence of the KASP marker allele linked to resistance, the validation panel was grouped into four haplotype groups (H1 to H4) (Table S10). The H1 group (*n* = 58) had resistant alleles for both QTL, the H2 group (n = 34) had the resistance allele for the 6DL QTL only, the H3 group (*n* = 20) had resistant allele for the 6DS QTL only, and the H4 group (*n* = 63) lacked both resistant alleles.

The H1 group had a significantly lower mean DBI (5.16%) than the H2 group (14.55%), while H2 group had a significantly lower mean DBI than both H3 (27.26%) and H4 (38.01%) groups (Fig. 4B). This result indicates that the combined effect of the two QTL is greater than that of the *Qdb.ssdhui-6DL* QTL only. The mean DBI of the H3 group was lower than that of H4 group, but the difference was not statistically significant (Fig. 4B). This result suggests that the effect of the *Qdb.ssdhui-6DS* QTL alone may be small, but the precision of the assessment could have been affected by the smaller number of lines in the H3 group compared to other haplotypes.

## Discussion

Using a bi-parental population, the QTL for DB resistance in cultivar UI Silver were mapped. DBI was highly heritable in this population, with the broad sense heritability exceeding 0.86 (Table 1). Correlations of DBI across the three evaluation years ranged from 0.68 to 0.93. Similar findings of high trait heritability and across-year correlations were reported for DBI by others (Wang et al. 2019; Gordon et al. 2020; Muellner et al. 2021).

### QTLs for DB resistance in resistant cultivar UI Silver

The present study indicates that UI Silver possesses two QTL for DB resistance. *Qdb.ssdhui-6DL* was detected in all datasets and explained a larger portion of the phenotypic variation for DB resistance compared to QTL *Qdb.ssdhui-6DS*. The two QTL together contributed a higher level of resistance than 6DL QTL alone in both the bi-parental and the validation panel although the effect of the 6DS QTL alone was not significant in the validation population. This result could be related to the small sample size available to test the effect of the 6DS QTL alone, as only 20 out of the 175 lines in the validation population had this haplotype. Some outliers with the 6DS + and 6DL + haplotype were observed to have a high DBI in both the mapping and validation populations (Fig. 3 and Fig. 4). Likewise, a few of the resistant lines did not have the marker alleles of both QTL. This observation suggests that there might be additional QTL/genes in either UI Silver or Shaan89150 contributing to susceptibility or suppressing one or two of the QTL identified. A similar explanation may apply to the validation panel. The KASP marker alleles for the 7DS QTL could not distinguish the two parents of the SSDH population and were infrequent in the validation panel (J. Chen, unpublished data).

Although the precision of QTL mapping was affected by the relatively small mapping population size of 135 lines and the limited recombination characteristic of DH lines, the two QTL identified were validated with the panel of regionally adapted lines and cultivars. To further explore the genetic control of the two QTL and better understand the genetic architecture of DB resistance in UI Silver, it will be essential to create fine-mapping populations using selected DH lines that target each of the QTL. Additionally, next-generation sequencing data from the two parents may be valuable for dissecting the resistance mechanism in UI Silver.

UI Silver originated from a BC_1_F_6_ line of the backcross IDO498*2/UT944157. IDO498 is a hard red winter wheat breeding line derived from the pedigree Turcikum 57/3*Manning and was developed by the University of Idaho Aberdeen Wheat Breeding Program. ‘Manning’ (Dewey 1981) is a hard red winter wheat cultivar developed by the Utah Agricultural Experiment Station with the pedigree Delmar / PI 178383 // Columbia /4/ Delmar /3/ UT 175–53 // Norin 10 / Brevor. UT944157 is a hard white winter wheat breeding line developed by the Utah State University Wheat Breeding Program that is a sib-selection of the hard white winter wheat cultivar ‘Golden Spike’ (Hole et al. 2002) with the pedigree Arbon / Hansel /4/ Hansel /3/ Cltr14106 / Columbia /2/ McCall. ‘Hansel’ has pedigree Delmar / PI 178383 // Columbia (Dewey 1981). PI 178383 appears in multiple generations of the UI Silver pedigree and is likely its source of DB resistance. PI 178383 is a known carrier of the bunt resistance genes *Bt8*, *Bt9, Bt10* (Menzies et al. 2006; Steffan et al. 2017; Lunzer et al. 2023a). *Bt9* and *Bt10* have been previously associated with resistance to both CB and DB in wheat (Menzies et al. 2006; Steffan et al. 2017; Wang et al. 2019; Gordon et al. 2020).

The marker interval of *Qdb.ssdhui-6DL* identified in UI Silver overlaps with that of a DB QTL previously identified in the resistant line ‘IDO835’ (Wang et al. 2019) and a CB QTL discovered in the resistant landrace ‘PI 166910’, the source of *Bt11* (Lunzer et al. 2023a) (Table 4). This region has been associated with the resistance gene *Bt9*, as described in Wang et al. (2019) and Lunzer et al. (2023a), suggesting that *Qdb.ssdhui-6DL* may either carry *Bt9* or is closely linked to it.

**Table 4:** The two identified QTL and their map and physical positions in relation to previously reported QTL for resistance to dwarf bunt (DB) and common bunt (CB)

Chr	Position (cM)	Position (Mbp)	Trait	Marker system	Population	Resistant parent	Resistance source	Gene	References
6DS	19.3	1.76 − 7.75	CB	SSR	DHLs	AC Taber	PI 178383	*Bt10*	Menzies et al. (2006)
6DS	9.7	1.76 − 7.75	CB	SSR and DArT	DHLs	AC Cadillac	BW553	*Bt10*	Singh et al. (2016)
6DS	0 − 3.7	1.4 − 2.1	DB	90 K SNP	DHLs	UI Silver	PI 178383	*Bt10*	This study
6DL	124.5 − 132.5	480.7 − 490.7	CB	SSR and DArT	DHLs	PI 554099	PI 178383	*Bt9*	Steffan et al. (2017)
6DL	87.45 − 89.01	487.2 − 492.6	DB	90 K SNP	DHLs	IDO835	PI 178383	*Bt9*	Wang et al. (2019)
6DL	13.9	492.6 − 495.2	CB	25 K SNP	RILs	PI 166910	PI 166910	*Bt11*	Lunzer et al. (2023a)
6DL	15.9 − 19.1	492.5 − 494.6	DB	90 K SNP	DHLs	UI Silver	PI 178383	*Bt9*	This study

Similarly, the marker interval of the *Qdb.ssdhui-6DS* QTL overlaps with a CB QTL found in the resistant line ‘AC Taber,’ which carries *Bt10* (Menzies et al. 2006), and in the resistant line ‘AC Cadillac’ (Singh et al. 2016). This interval also aligns with a locus identified in an association mapping study using a diverse wheat panel from the USDA-ARS National Small Grain Collection (Gordon et al. 2020). Thus, *Qdb.ssdhui-6DS* may carry either *Bt10* or other closely linked resistance genes.

The present study also developed and validated KASP markers that can be used in MAS to improve for both CB and DB resistance. The additive effect of *Qdb.ssdhui-6DL* and *Qdb.ssdhui-6DS* holds promise for breeding high levels of resistance by selecting lines with favorable alleles at both QTL. Since *Qdb.ssdhui-6DS* QTL alone did not significantly decrease DBI in the validation panel, selecting for the *Qdb.ssdhui-6DS* QTL alone may not be useful.

### Source of resistance and resistant cultivars

Developing DB resistant wheat for dryland winter wheat production is a primary focus in the USU and UI breeding programs (Hole and Clawson 2023). Many dryland production areas in the Intermountain West experience frequent and prolonged snow cover during the seedling stage, favoring *T. controversa* spore germination. Severe epidemics occurred in the 1940s and 1950s, causing significant losses in grain yield and quality (Tyler and Jensen 1958; Goates 1996; Borgen and Davanlou 2001). Since the 1960s, bunt diseases have been controlled through the deployment of resistant cultivars and the use of fungicides (Muhae-Ud-Din et al. 2020; Lunzer et al. 2023b).

Two known source of resistance, ‘PI 178383’ and ‘PI 476212’, have been widely used in cultivar development in the USU and UI programs. PI 178383 is a DB-resistant landrace that was collected in Hakkari, Turkey and deposited in the National Small Grain Collection (NSGC) in 1949. It is tall with weak straw and brown chaff, and it also has adult plant resistance to stripe rust and resistance to Russian wheat aphid (*Diuraphis noxia*) (https://npgsweb.ars-grin.gov/gringlobal/search). PI 178383 is present in the pedigrees of several wheat cultivar releases, including ‘Hansel’ (Dewey 1975), ‘Manning’ (Dewey 1981), ‘Deloris’ (Hole et al. 2004), ‘Promontory’ (Hole et al. 1995), ‘Utah 100’ (Hole et al. 1997), ‘Weston’ (Morris and King 2002), ‘Golden Spike’ (Hole et al. 2002), ‘UI Darwin’ (Souza et al. 2008a), ‘Juniper’ (Souza et al. 2008b), ‘UI Silver’ (Chen, 2010), ‘UI SRG’ (Chen et al. 2012), ‘Lucin CL’ (Hole 2011), and ‘UI Sparrow’ (Chen et al. 2018). These cultivars are among the 53 resistant lines (DBI < 10%) that have either resistance alleles at the 6DL QTL alone or at the 6DL plus 6DS QTL, as characterized by marker haplotypes (Table S10).

‘PI 476212’, a soft red winter wheat deposited in the NSGC in 1982, is also highly resistant to DB,CB, and Russian wheat aphid (Sunderman et al. 1986). A tall line with weak straw and brown chaff, PI 476212 is also tolerant to snow mold (*Typhula idahoensis*) but susceptible to leaf and stripe rusts (https://npgsweb.ars-grin.gov/gringlobal/search). PI 476212 contributed to three UI cultivar releases, Blizzard (Sunderman et al. 1991), DW (Souza et al. 2004), and Bonneville (Souza et al. 1995) (Table S10). These cultivars lack the resistance allele at the 6DL QTL, but they do have the same marker haplotypes as ‘IDO444’, which was the resistant parent in a previous bi-parental mapping study that identified a QTL for DB resistance on chromosome 7DS (Chen et al. 2016). Bonneville and Blizzard have QTL associated with CB resistance on chromosome arms 1BS, 1AL, and 7AL, with DB resistance on chromosome arms 1AL, 7AL, and 7DS (Muellner et al. 2020). These results indicate that PI 476212 may represent a different source of resistance from PI 178383.

The present study also identified a few resistant cultivars (DBI < 10%) released from WSU program, such as ‘Luke’ (Peterson et al. 1974), ‘Eltan’ (Peterson et al. 1991), ‘Bruehl’ (Jones et al. 2001), and ‘Otto’ (Carter et al. 2013). PI 178383, the last parent in Luke’s pedigree, is likely the source of DB resistance in Luke. Luke was the last parent of Eltan, and Eltan was subsequently the last parent of Otto and Bruehl. While these cultivars lacked resistance alleles at the 6DL and 6DS QTL, they do share the same marker haplotypes associated with the DB resistance as of IDO444, which contains the resistance allele at the 7DS QTL (Chen et al. 2016). Thus, PI 178383 likely has multiple DB resistance genes. Bunt differential lines *Bt8, Bt9*, and *Bt10* were derived from the cross between PI 178383 and the highly susceptible cultivar ‘Elgin’ (Goates 2012), supporting the hypothesis of multiple resistance genes in PI 178383. Byusing PI 178383 as a resistant parent one can select lines containing resistance allele at the 6DL and 6DS QTL plus the 7DS QTL depending on the selection pressure. DB is the main resistance breeding target for the dryland winter wheat breeding program at USU (Hole and Clawson 2023), whereas breeding for stripe rust resistance has been a goal in WSU winter wheat breeding program (Chen 2020). At the UI breeding program, DB is the major disease resistance target for dryland winter wheat breeding and stripe rust resistance is the main breeding objective for irrigated winter wheat. Adapted cultivars and lines characterized in the present study that have resistance alleles from PI 178383 and PI 476212 can be used as parents in breeding programs to generate new cultivars with DB and CB resistance using molecular MAS.

## Conclusion

The present study identified two QTL for DB resistance on chromosomes 6DL and 6DS. Resistance alleles were identified in the winter wheat cultivar UI Silver, the resistant parent in the SSDH bi-parental mapping population. KASP markers associated with the two QTL were developed and validated in a winter wheat validation panel. *Qdb.ssdhui-6DL*, located on chromosome 6DL (492.55–494.66 Mbp), was the primary QTL controlling DB resistance in the resistant cultivars and lines in the validation panel, while QTL *Qdb.ssdhui-6DS* positioned on chromosome 6DS (1.40–2.15 Mbp), was less effective but had an additive effect with *Qdb.ssdhui-6DL* for enhanced DB resistance. These findings contribute to the wheat community’s understanding of the genetic architecture of DB resistance. The KASP markers and characterized resistant lines identified in the present study are valuable resources for generating wheat cultivars with DB resistance.

## Supplementary Information

Below is the link to the electronic supplementary material.

Supplementary file1 (XLSX 14976 KB)

Supplementary file2 (CSV 27185 KB)

## Data Availability

All data generated during this study, including the raw 90 K SNP data, are included in this published article and its supplementary information files.
